# An Investigation Into the Role of English as a Foreign Language Teachers’ Self-Efficacy in Their Organizational Commitment

**DOI:** 10.3389/fpsyg.2022.894333

**Published:** 2022-05-20

**Authors:** Yuan Gao

**Affiliations:** School of Humanities and Law, Fuzhou Technology and Business University, Fuzhou, China

**Keywords:** self-efficacy, organizational commitment, foreign language learning, organization success, committed teachers

## Abstract

The way EFL students experience the process of learning has always been of utmost importance since it tremendously affects the amount of learning and received pleasure throughout this process for both teachers and students. From this aspect, both self-efficacious and committed teachers make a contribution to their organization’s success. Even though many studies have been conducted about teachers’ self-efficacy and their organizational commitment, a few of which concentrate their attention on the link between these two variables. To fill this gap, this review paper provides a glimpse at the underlying roles of teacher self-efficacy and their organizational commitment. Furthermore, it presents the definitions and applications of each construct. Finally, some implications and future recommendations are put forward to avid scholars.

## Introduction

Owing to technology spreading everywhere which causes new types of needs and due to obviating those needs, it inevitably happens that all people including teachers need to find a way to make a cordial atmosphere in their work lives so as to find the peace that has a positive impact on their wellbeing otherwise they cannot feel satisfied with their jobs. It, therefore, is necessary to be relaxed when confronting complicated problems and see them as a challenge, not a blockage. As a result of which self-efficacious teachers seem to have higher wellbeing and are more committed to where they work. In the educational domain, teachers play a paramount role since students’ learning process and their motivation can be enhanced and the way the process of learning would be facilitated is directly aligned with the way students are treated by their teachers. Due to the fact given, teachers’ self-efficacy and its impact on their organizational commitment have been highlighted. More commitment leads to taking the responsibility of having a more productive class where students’ linguistic talents can be cultivated and their ideas can be expressed through showing their language skills which can be regarded as teachers’ commitment.

Since teachers play a pivotal role in the educational milieu, their capability is of utmost importance. According to social cognitive theory (Bandura, 1977, 2006), self-efficacy in instructional contexts is characterized by the belief in being able to deal with behavioral and learning problems efficiently throughout the class. It is also conceptualized as the extent to which a teacher believes in his ability to arrange and implement the amount of action needed for achieving a certain teaching task in a specific situation (Tschannen-Moran et al., 1998). According to Bandura (1977), self-efficacy is concerned with how far people believe and perceive their competencies. Some variables are said to be correlated with teachers’ self-efficacy, such as crowning academic achievements, higher levels of commitment, and efficacious teaching methods (Ware and Kitsantas, 2007). Teachers’ self-efficacy also helps teachers to be better and they can be more satisfied with their jobs (Skaalvik and Skaalvik, 2007).

Teachers’ organizational commitment, on the other hand, is conceptualized as a sense of belonging to the school a teacher works in Reyes (1990). However, emphasis should be placed on the fact that few studies have been carried out to stress the significance of teachers’ self-efficacy and its relevance to their organizational commitment in instructional domains. In addition, hardly ever has a study conducted in the context of China in which the impact of teachers’ self-efficacy on organizational commitment has been discussed. Moreover, what makes it an important issue to be probed is its important role in teachers’ motivation and how committed they feel to the school or the organization where they work. This study hence aims to emphasize the relationship between EFL teachers’ self-efficacy and their organizational commitment. In an attempt to do that, first of all, two variables have been defined and their importance has been highlighted. Teaching efficacy then has been dealt with as well as its link with EFL teachers’ organizational commitment, and some implications and further recommendations for future studies have been finally put forward.

## Background of Research

### EFL Teachers’ Self-Efficacy

Self-efficacy is conceptualized as one’s beliefs about his capacities to acquire specific achievements (Tschannen-Moran et al., 1998). Many factors have been influenced by self-efficacy, for example, the way they put effort into practice to reach their goals, how resilient and persistent they are while faced with a problem, how tolerant they are when facing a tough situation like a failure, how much stress is experienced by them when they are under pressure (Bandura, 1977). Teacher self-efficacy has drawn attention to itself since it can tremendously affect schools and students’ lives. Having this in mind, teacher self-efficacy is defined as teachers’ beliefs about the extent to which they can impact their students’ performance. Therefore, the amount of effort teachers put into teaching, the way they set goals and how realistic they are, the amount of enthusiasm and aspiration they show in their job are all affected by teachers’ sense of efficacy. Teachers who have a higher sense of efficacy are thought to be more passionate and more committed toward their job and they are more likely to stay as teachers for the rest of their lives (Tschannen-Moran et al., 1998). Given that, self-efficacy is one of the crucial constructs for teachers through which many positive behaviors in teaching can arise, contributing to prosperous outcomes for students (Henson et al., 2001). Teachers with higher efficacy are said to persist more when encountering problems and dealing with less motivated students. Their effective teaching also affects such students’ development (Gibson and Dembo, 1984). Moreover, they welcome novel experiences and show more commitment (Guskey, 1988; Coladarci, 1992).

Based on Martín (2000) and Bandura (2001), self-efficacy is the determiner of motivation, feelings, thoughts, and actions. Teachers, hence, try not to do the tasks that are beyond their capabilities, instead they do the ones that are controllable. Given that, self-efficacy is linked with job stress since some of the outcomes of stress, such as not being satisfied with jobs, physical fatigue, and a drop in organizational commitment, can be mitigated through self-efficacy (Jex and Bliese, 1999). It was also claimed that both feeling anxious and depressed can be decreased (Beas and Salanova, 2006), as well as burnout (Salanova et al., 2000, 2001). The mediating role of self-efficacy is also stressed in developing both motivational and erosion processes (the presence of low levels of professional self-efficacy) of burnout and engagement at work (Salanova et al., 2011; Vera et al., 2012). As a result of which self-efficacy affects the way the environment is understood which means without self-efficacy and these beliefs, teachers could not have been persevered and motivated when facing hardships. Thereby, according to Bandura (2001), it is self-efficacy which paves the way for teachers to see problems as stepping stones or controversies, not the events that are not controllable. The difference between these two views is that when problems are addressed as challenges, people believe that solutions can be raised even for the perennial ones, while when problems are seen as uncontrollable events or hindrances, hardly ever do people come to the recognition that the problems would be soluble and can be tackled. Moreover, those with lower self-efficacy have been perceived to feel more pessimistic about their performances and achievements and accordingly they may suffer from depression and anxiety (Schwarzer, 1999). While those who have higher self-efficacy are more likely to feel more positive that impacts their level of dedication, perseverance, and vigor (Llorens, 2004; Bakker et al., 2006, 2007; Mauno et al., 2007).

Furthermore, it has been argued that both engagement and burnout are affected by self-efficacy (Cherniss, 1993; Llorens et al., 2005, 2007; Salanova et al., 2011; Vera et al., 2012), in that teachers with higher self-efficacy are more willing to engage in efficacious teaching and also involve the students; whereas those with lower levels are prone to feel burnout and they are mostly dissatisfied with their jobs. Self-efficacy should be different from academic self-concept that is concerned with the questions that goes as follows: Am I good at what is going to be done? Skaalvik and Skaalvik (2007). Bandura (1977) claimed that self-efficacy stems from prior mastery experiences with similar kinds of tasks, for example when a teacher observes other teachers who master similar controversies; persuading verbally, for example, when a teacher is socially supported by his colleagues and the school authorities; and physiological arousal, for example, a teacher feels that his heart is pounding while faced with a challenging situation. Out of which the most effective source is the first one, prior mastery experiences. It is pinpointed that both job satisfaction and exhaustion are positively and negatively correlated with self-efficacy, respectively. Job satisfaction which is defined as the extent to which people judge and evaluate their job (Weiss, 1999) is positively aligned with self-efficacy according to a study conducted by Skaalvik and Skaalvik (2016). Since it develops a sense of autonomy in people, contributing to a higher self-esteem. On the other hand, emotional exhaustion and time pressure are negatively related to self-efficacy. Emotional exhaustion is defined as sapped energy, chronic fatigue, debilitation, and feeling of being worn out (Pines and Aronson, 1988; Schwarzer et al., 2000). It is regarded as the factor affecting the most on burnout and it is rooted in enduring stress which is relevant to their profession (Jennett et al., 2003).

Based on what Maslach and Jackson (1981) put forward, burnout is characterized as a syndrome comprising emotional exhaustion, depersonalization, and reduced personal accomplishment. As shown, burnout is negatively linked with teacher self-efficacy (Brouwers and Tomic, 2000; Skaalvik and Skaalvik, 2007) and job satisfaction (Skaalvik and Skaalvik, 2010). Based on Tschannen-Moran et al. (1998) and Tschannen-Moran and Woolfolk Hoy (2001), self-efficacy comprises three classifications: efficacy in engaging the students, efficacy in educational techniques, and efficacy in managing the class. The first category refers to the amount students are actively engaged in the class activities which was confirmed to have a direct relationship with both teachers’ self-efficacy and the way teaching strategies are provided in the class and how far students are encouraged by their teachers to indulge in the activities. With respect to the second category, the way the subject matter is presented in the class highly affects the students’ performance and teachers’ self-efficacy has been found to bear a positive impact on it. The third classification that is concerned with the way the class is managed has been thought to have a significant correlation with the amount of self-efficacy in teachers. The higher the self-efficacy is, the better the class can be managed which contributes to students’ enthusiasm in learning a new language. Hence, there is a positive correlation between self-efficacy and psychological wellbeing (Fathi et al., 2020). Psychological wellbeing is defined as having autonomy, growth mentality, building up positive relationships with others, setting a goal in life, and self-acceptance. Similarly, teachers’ characteristics and their psychological wellbeing have a positive effect on teachers’ performance and the way the materials are presented in the class (Fathi and Derakhshan, 2019). According to a study, the relationship between teachers’ personal characteristics and their effectiveness has been probed. It turned out that there is a higher possibility for self-efficacious teachers to act more effectively, teaching a second language (Klassen and Tze, 2014). Teachers’ self-efficacy was discovered to be an important predictor of job satisfaction as well (Türkoglu et al., 2017). Another study indicated that teachers with higher self-efficacy are more likely to endure the problems throughout the class and put up with a lot (Wyatt, 2012).

### EFL Teachers’ Organizational Commitment

Teaching has been perceived as a job that is rewarding, yet stressful (Liu and Onwuegbuzie, 2012; McInerney et al., 2018). Commitment has been claimed to be one of the most significant characteristics one can develop in his life. In the instructional domain, teacher commitment can be explored from different aspects. Committed teachers are supposed to have strong beliefs in the goals which are related to teaching, to be passionate to put efforts into practice to reach those goals, and be persistent to be engaged with it and following it (Firestone, 1996). According to Firestone and Pennell (1993), teachers’ commitment differs from person to person, regarding the aims they are committed to, meaning that teachers can make a commitment to the way they teach, to their organizations, or to their students. Lee and Smith (1996) showed that the more committed the teachers are and the more they can take responsibility for students’ learning process, the more students achieve, considering the academic goals they have set. It has been argued in other studies that teachers’ commitment is affected by the levels of student achievement. The higher the achievements of the students are, the more willing teachers to shoulder the responsibility of teaching (Firestone and Rosenblum, 1988; LeCompte and Dworkin, 1991).

Likewise, teaching commitment was conceptualized as a sense of belonging to the school a teacher works in (Mowday et al., 1979; Reyes, 1990). Three factors should be taken into consideration with regard to teaching commitment. Firstly, each school or educational organization has its own objectives and values, the way teachers approve and feel loyal to them can be regarded as a teachers’ organizational commitment. Secondly, making efforts and being dedicated on behalf of the school and the organizations also falls under the category of teaching commitment. Thirdly, how far teachers are inclined and passionate to stay with the organization is another emblem of teachers’ organizational commitment. With respect to the above-mentioned points, two different aspects of commitment can be taken into account: professional commitment and organizational commitment (Billingsley and Cross, 1992; Firestone and Pennell, 1993).

Students’ performance is tremendously affected when their teachers are actively engaged in the class and dedicated to what they are expected to do in the class, so it is what causes the learning process to be improved (Shukla, 2014). To put it simply, to enhance students’ success and facilitate the process of achieving their goals, schools play a vital role and their success is highly dependent on the amount of commitment that teachers make (Lee et al., 2011). As Rosenholtz (1991) stated high performance and commitment to work and the organization are promoted by intrinsic motivation, meaning that teachers who are intrinsically motivated are unlikely to shrink their responsibilities and they are more committed to their work and to where they work. As a consequence, many factors including teachers’ quality of performance, their inclination to outlast in their profession, being tolerant and satisfied with the organizational aims, dedicating more time and perseverance to their job, and their job satisfaction are linked with the commitment of teachers (Yousef, 2000).

Cooperation with both colleagues and parents is also said to surge if the high sense of self-efficacy can be found in teachers, resulting in an effective and cooperative working ambiance where teachers can readily talk about their work problems (Hoover-Dempsey et al., 1992; Caprara et al., 2006). Teachers’ level of commitment to their job and to their students’ learning has been confirmed to be relevant to the amount to which they believe in their capabilities. If teachers strongly believe in themselves, students’ learning will successfully be enhanced (Bandura, 1977). As was asserted by Tschannen-Moran and Woolfolk Hoy (2001) teaching passion, being committed to teaching, and staying in the same workplace for long have been influenced by teacher efficacy. Teachers with stronger self-efficacy and more commitment have a tendency to help their students more to be successful, in comparison to the teachers who do not have faith in their abilities which was the way teacher efficacy was defined. Therefore, it has been argued that class activities are better planned and students with difficulties in learning are better helped, and more appropriate teaching materials are found when teachers are self-efficacious. Teachers with greater confidence are reported to deal with teaching situations that challenge them through the class more easily. They are also more responsible to tackle their psychological problems since many problems may arise when teaching; even though some of them might not be massive, they would disturb the teachers and they could not focus their attention on what they do for a specific amount of time.

### The Relationship Between EFL Teachers’ Self-Efficacy and EFL Teachers’ Organizational Commitment

In the educational domain, teachers are proven to bear a striking impact on students’ performance. As teachers’ self-efficacy has been defined, it is the extent to which teachers believe that students’ performance can be affected by them; therefore, they endeavor to make their teaching as effective as they can and they are willing to find a solution to the problems faced through this process, either perennial or minor ones. In other words, teachers with higher self-efficacy will assuredly be very likely to take the responsibility of their teaching and be more committed, contributing to a surge in students’ confidence and resilience and the way they can cope with the problems with which they are faced. Likewise, when students are more resilient and self-assured, it influences both their learning process and social life in that they learn to believe in themselves to have a voice both in the instructional context and in society. They are also less reluctant to feel shattered and diffident when encountering difficulties in their learning process and in society. To put it simply, they know how to pull themselves together and not lose hope since success takes energy, perseverance, and persistence and as a result of which sometimes students fail to achieve their desired goals and may feel defeated. From a closer aspect, learning a language is a step-by-step commitment which means a long-term commitment should be made to learn different skills of a language; hence, for a EFL student to be committed in the whole process, there should be a role model like teachers by whom they are influenced.

Organizational commitment, on the other hand, can be shown in teachers’ effective way of teaching, encouraging students to implement their educational duties in a good way, cultivating students’ natural talents if their linguistic intelligence is outstanding, making them motivated to take the responsibility of their own learning, helping students to heighten their self-esteem in order to enjoy the process of learning more. As mentioned above, self-efficacy is negatively linked with burnout since teachers with lower levels of self-efficacy do believe in their capacities, resulting in good performances of their own and their students. This trend is reversed for engagement as self-efficacy and engagement are positively correlated. In other words, people with higher levels of self-efficacy are more engaged in the class, leading to their higher wellbeing. It is rooted in the fact that self-efficacious teachers are more satisfied with their jobs which are extremely demanding and to a great extent stressful.

Additionally, high levels of stress and heavy workload cause teachers to feel less engaged, and as a result of which burnout arises. That is the reason why an increasing number of teachers leave their jobs on a yearly basis. It is where the importance of self-efficacy in teachers should be strengthened to inform the authorities of their crucial responsibility and how significant this trait is and how it can be associated with the levels of organizational commitment. It turns out that there should be an underlying reason behind the commitment that can be found in teachers. It is, therefore, of paramount importance to consider self-efficacy as a variable that plays a predictive role in teachers’ level of commitment. As has been shown, teachers with higher levels of wellbeing are more liable to feel committed to what they do throughout the class and the amounts of their job satisfaction. Therefore, it can be vividly apparent that teachers with lower self-efficacy magnify the problems and see the shortcomings as hindrance; with this in mind, they are not inclined to think about the possible solutions so as to resolve the problems and it is when they feel demotivated and devastated, leading to being bereft of what they do. They act as though their mindsets are hardwired and the way they think of the situations is unalterable. Considering the above example, it is easier said than done to perceive teachers since teachers have always been pressurized with heavy workload, coping with stress, and come up with new ideas for better teaching, they may feel the way described every now and then; consequently, higher self-efficacy helps teachers to keep their composure and feel more committed as they do their jobs.

From an organizational aspect, teachers with high self-efficacy, as explained above, are more likely to respect the objectives and the atmosphere of the place they live. They are, additionally, more willing to work in harmony with their colleagues and be dedicated to their job and to the organization in which they work. It also affects the quality of the way they teach. The higher self-efficacy, the more committed they are to their job and the more they stay in the school or organization where they work. Job satisfaction has also been found to increase if teachers are more committed. As a result, according to some studies, there is a positive correlation between teacher self-efficacy and teacher commitment (Ross and Gray, 2006; Ware and Kitsantas, 2007; Klassen and Chiu, 2011; Canrinus et al., 2012).

## Implications in the Research

The aim of this study was to stress the association between teachers’ self-efficacy and their organizational commitment. As shown, there is a positive correlation between these two variables and a rise in one of which results in an increase in another one. Therefore, both teachers and educational authorities benefit from such studies. In-service courses should be planned for teachers to remind them of their significant role in the instructional areas and how students’ academic life can be impacted by them. On the other hand, educational authorities which are in charge of strengthening educational infrastructures are said to equip teachers with high self-efficacy and teach them how it can be increased to affect both their mental wellbeing and effectiveness in their classes. Similarly, this study is also of great benefit for Language educators since not only should they look for well-educated experienced teachers who know how to teach properly, but they should care about teachers with higher self-efficacy who are more likely to be committed to where they work. Experience does not just consist of teaching years; however, their mental health should be taken into account.

It should also be beneficial for both students and parents because they are supposed to be cognizant of the fact that good teachers are not just knowledgeable, but their mental wellbeing has a considerable effect on the way students are encouraged to learn a new language. Teachers themselves had better be aware of the fact that it is not just their knowledge that distinguishes them from their colleagues but their mental health is what makes a difference as well. They, thus, need to care more about their health.

Future studies relevant to these topics can be categorized into several groups. Firstly, longitudinal studies with better quality can be conducted since it takes time to consider all the traits that teachers with higher self-efficacy show. Secondly, the resources which help to increase self-efficacy should be dealt with and the way they can be applied in classes should be taken into consideration. Thirdly, other types of commitment had better be studied as well as their roles in the educational domain, professional commitment, to name a few.

As Skaalvik and Skaalvik (2007) stated self-efficacy is divided into 3 categories: class management, students’ involvement, and educational strategies, so different studies can be conducted to find the relevance of each aspect of self-efficacy to other important variables as each of them seems to be extremely important on its own and plays a radical role in how teachers act in the classroom. Likewise, another variable of this study, organizational commitment, can deeply be investigated in that commitment as a personality trait and its relationship with self-efficacy can be studied. To put it simply, to what extent a committed person can be a committed teacher, considering his vocational goals. In conclusion, there should be some studies to work on the association between these two variables since they play a key role in both teachers’ and students’ success.

## Author Contributions

The author confirms being the sole contributor of this work and has approved it for publication.

## Conflict of Interest

The author declares that the research was conducted in the absence of any commercial or financial relationships that could be construed as a potential conflict of interest.

## Publisher’s Note

All claims expressed in this article are solely those of the authors and do not necessarily represent those of their affiliated organizations, or those of the publisher, the editors and the reviewers. Any product that may be evaluated in this article, or claim that may be made by its manufacturer, is not guaranteed or endorsed by the publisher.
